# Reciprocal Dynamics of Metabolism and mRNA Translation in Tumor Angiogenesis

**DOI:** 10.3390/ijms252011284

**Published:** 2024-10-20

**Authors:** Jacopo Lidonnici, Roxana E. Oberkersch

**Affiliations:** 1 Department of Surgery, Oncology and Gastroenterology, Section of Oncology and Immunology, University of Padova, 35128 Padova, Italy; jacopolidonnici@gmail.com; 2 Department of Oncology, University of Torino, 10060 Candiolo, Italy

**Keywords:** tumor vasculature, endothelial cell metabolism, mRNA translation

## Abstract

Angiogenesis, the process of formation of new blood vessels from pre-existing vasculature, is essential for tumor growth and metastasis. Anti-angiogenic treatment targeting vascular endothelial growth factor (VEGF) signaling is a powerful tool to combat tumor growth; however, anti-tumor angiogenesis therapy has shown limited efficacy, with survival benefits ranging from only a few weeks to months. Compensation by upregulation of complementary growth factors and switches to different modes of vascularization have made these types of therapies less effective. Recent evidence suggests that targeting specific players in endothelial metabolism is a valuable therapeutic strategy against tumor angiogenesis. Although it is clear that metabolism can modulate the translational machinery, the reciprocal relationship between metabolism and mRNA translational control during tumor angiogenesis is not fully understood. In this review, we explore emerging examples of how endothelial cell metabolism affects mRNA translation during the formation of blood vessels. A deeper comprehension of these mechanisms could lead to the development of innovative therapeutic strategies for both physiological and pathological angiogenesis.

## 1. Introduction

Cancer, defined as a large group of diseases that can start in almost any organ or tissue of the body when abnormal cells grow uncontrollably, is the second worldwide cause of death after ischemic heart disease but will likely become the first in 2060 [1]. The International Agency for Research on Cancer (IARC), part of the World Health Organization (WHO), estimates that the number of new cancer cases worldwide will increase by 63.4% from 2020 to 2045. In 2020, 2.7 million people in the European Union were diagnosed with cancer, and another 1.3 million people lost their lives to it, including over 2000 young people [2]. For this reason, biomedical research on the histological features, molecular mechanisms, and risk factors underlying tumor malignancies has extensively been made over the past century, and during the last two decades it has become clearer that, although mainly deriving from genetic and epigenetic alterations influencing the expression and activity of proto-oncogenes and tumor suppressor genes, cancer onset and progression are also deeply influenced by the surrounding vessel’s formation [3,4].

Once a neoplastic mutation has occurred in somatic cells, an avascular phase of tumor growth follows. Tumor cells are supplied by diffusion, and tumor growth is arrested at a size of 1–2 mm [3]. The following stadium of “tumor dormancy” can last up to years. When pro-angiogenic factors overcome the effect of angiostatic molecules, the tumor acquires an angiogenic phenotype that leads to the formation of new blood vessels in a process known as angiogenesis. As angiogenesis is one of the hallmarks of cancer, a number of anti-angiogenesis drugs have been FDA- and EMA-approved and are being used in cancer treatment, and a number of other agents are in different stages of clinical development or in preclinical evaluation [5,6]. However, conventional pharmacologic anti-angiogenesis strategies based on targeting and blocking the activity of pro-angiogenic factors (e.g., bevacizumab, sunitinib and pazopanib) have shown to be insufficient in most cases of cancer [7]. For this reason, new molecular strategies have been proposed to target vessel formation based on reactive oxygen species (ROS) generation, beta-adrenergic drugs, DNA repair controllers and endothelial cell (EC) metabolism [6,8,9]. While the modulation of ROS and ROS-mediated DNA damage controllers as alternative approaches to target tumor angiogenesis are still under study [6,10], now it has become clear that ECs, often differently from other cell types, rely on distinct metabolic pathways to survive and form new blood vessels and that the manipulation of EC metabolic pathways alone (even without changing angiogenic signaling) suffices to alter vessel sprouting. Furthermore, perturbations of these metabolic pathways can underlie the excess formation of new blood vessels in cancer [11,12,13,14,15]. Nonetheless, it is essential to take into account key considerations when developing anti-angiogenic strategies that focus on a single metabolic enzyme: (i) to date, the function of only a few key metabolic enzymes in ECs has been characterized, limiting our view of EC metabolism [11,13,14,16]; (ii) tumor microenvironment converts normal ECs to tumor endothelial cells (TECs) and induces a specific EC metabolic reprogramming [17]; and (iii) a new mechanism of resistance to anti-angiogenic therapies was described that involves a metabolic symbiosis between tumor and surrounding cells, which gives rise to metabolic heterogeneities [18].

**Figure 1:**
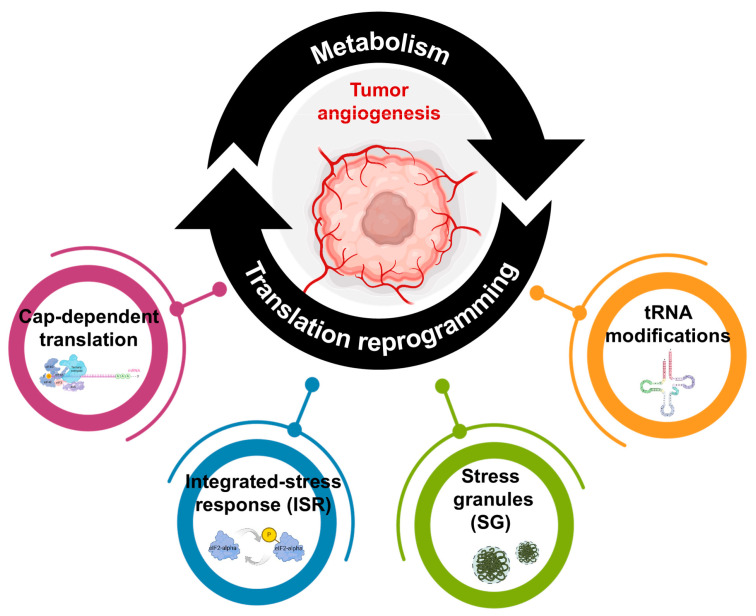
Crosstalk between endothelial cell metabolism and mRNA translation during tumor angiogenesis. The angiogenic activity of endothelial cells depends on their metabolic state, which influences the translational apparatus. Four translational actors—Cap-dependent regulation of translation, integrated stress response (ISR), stress granule (SG) formation and tRNA metabolism—are exquisitely controlled by metabolism and play a crucial role in endothelial cells (ECs), influencing vasculature formation. Some elements of the figure were created with BioRender.com.

The demonstration that protein concentration is determined by translational efficiency (TE) rather than messenger RNA (mRNA) abundance, and that changes in mRNA levels are evolutionarily neutral, represents a breakthrough in our understanding of gene expression [19,20]. This leads us to ask: might mRNA translation orchestrate the TEC metabolic program by modulating key enzymes, and could its modulation be a novel targetable angiogenic approach in tumor settings? Conversely, might different metabolic pathways rewired in a tumor context produce a pro-angiogenic phenotype through the modulation of translational machinery? To understand this complex crosstalk between metabolism and mRNA translation, we illustrate known examples of metabolic players whose translation is regulated temporally and sometimes spatially in tumor development and progression (Figure 1). In addition, in our concluding remarks, we will address some ideas and potential approaches for future research on the interplay between metabolic programs and translational regulation.

## 2. Metabolic mRNA Cap-Dependent Translation Modulation in ECs

Proteins are the ultimate products of genetic information flow, acting as its molecular effectors. The process of mRNA translation, or protein synthesis, is the most energy-intensive activity within the cell and is thus meticulously regulated at various levels. Typically, protein translation is divided into three main stages: initiation, elongation and termination. The rate-limiting step in protein synthesis is the initiation phase, where eukaryotic initiation factors (eIFs) assemble on the mRNA to commence translation [21].

Most eukaryotic transcripts have a 5′-terminal Cap (5′-Cap) formed by a 7-methylguanosine residue (m^7^G) linked by a 5′-5′-triphosphate bridge to the rest of the mRNA (Figure 1). This structure, in addition to promoting nucleo-cytoplasmic export and preventing transcript degradation, allows the anchoring of the initiation factor eIF4E [22]. The 5′-Cap binding protein (eIF4E) is associated with the mRNA template, the dead box RNA helicase (eIF4A) and a scaffold protein (eIF4G) form the eIF4F complex [23]. eIF4E represents a hub for the regulation of the entire translation apparatus. Under active translation conditions, various signaling pathways lead to the hyperphosphorylation of eIF4E binding protein (4EBP) and the release of sequestered eIF4E from the hypophosphorylated form. At the same time, the ternary complex (TC), composed of eIF2 (containing α-, β-, and γ-subunits), GTP and the initiator methionyl-tRNA (tRNA_i_^Met^) associates with the minor ribosomal subunit (40S) and other initiation factors such as eIF1, eIF1A, eIF3 and eIF5 to form the 43S pre-initiation complex (43S PIC) [24,25]. Subsequently, the association between the eIF3 belonging to the 43S PIC and the eIF4G scaffold belonging to the eIF4F complex drives the assembly of the 48S PIC complex. The association between eIF4G and the poly(A)-binding protein (PABP) linked to the 3′ poly(A) tail of the transcripts allows the circularization of the mRNA. The initiation phase ends when the tRNA_i_^Met^ is positioned on the start codon (AUG), the eIFs are released and the union with the large ribosomal subunit (60S) forms a translation-competent 80S ribosome [24,25]. Elevated levels of eIF4E have been positively correlated with VEGFA expression and tumor angiogenesis [21,26,27]. Furthermore, the pharmacological inhibition of eIF4E has also been shown to reduce angiogenesis in cancer contexts [28,29,30]. Therefore, targeting translation initiation could be a promising therapeutic strategy to attenuate the formation of cancer-associated blood vessels.

The main regulatory events leading to eIF4E activation are the eIF4E phosphorylation by the MAPK-interacting kinase (MNK) pathway and the hyperphosphorylation of 4EBP by the mammalian target of the rapamycin complex 1 (mTORC1) pathway [31] (Table 1). The activity of these pathways depends on permissive conditions, such as the presence of growth factors and nutrients. Recently, Oberkersch and colleagues observed how amino acid metabolism plays a key role in angiogenesis. In particular, in ECs, the transaminase-mediated aspartate metabolism triggers mTORC1 activation, which leads to the initiation of the translation of endothelial growth factor receptors crucial to supporting tumor angiogenesis [13] (Table 1). This suggests a close relationship between protein synthesis, angiogenesis and metabolic conditions.

Stress conditions, such as oxygen deprivation (hypoxia), reduce the global rate of mRNA translation [32]. In fact, hypoxia, a well-documented inducer of vessel formation, has been shown to suppress protein synthesis by blocking the three stages of translation: initiation, elongation and termination. Severe hypoxia (<0.05% O_2_), which activates the transient phosphorylation of eIF2α [33], blocks TC formation and slows the recruitment of ribosomes onto mRNA transcripts [34]. Moreover, hypoxia is capable of reducing overall translation without reducing ribosome density due to a disruption of the Cap binding complex eIF4F [35]. Furthermore, mRNA-to-protein translation elongation rates are significantly decreased due to elongation factor 2 (eEF2) inhibition by eEF2 kinase [36]. Also, during hypoxia, eukaryotic release factor 1 (eRF1) hydroxylation is decreased, inhibiting stop codon recognition and promoting more incidents of readthrough [37]. Although hypoxia leads to a general inhibition in translation, the consequence at the individual gene level is highly variable. This is due to the fact that 5′ and 3′ untranslated regions (UTRs) of mRNA play a significant role in regulating translation and mRNA half-life. They also allow transcripts to interact with specific RNA-binding proteins, which could confer their ability to maintain, or even increase, translation efficiency in spite of the overall inhibition [38]. Angiogenesis is also the result of a balance between stimulating and inhibiting factors, which are also under translational regulation [39]. Indeed, interferon γ activation of myeloid cells induces the assembly of the GAIT complex, which binds 3′UTR VEGFA mRNA elements and silences its translation. Under hypoxia, HLDA complex (hypoxia-induced hnRNP L-DRBP76-hnRNPA2/B1) binds GAIT element inducing VEGFA expression. Furthermore, fibroblast growth factor 1 (*FGF1*) gene, a known pro-angiogenic factor, has four alternative tissue-specific promoters, which results in mRNAs differing by their 5′UTR, suggesting specific translational regulation depending on the promotor usage [40]. Moreover, it has been shown that, during hypoxia, the eIF4F complex switches to a non-canonical form (eIF4F^H^), modifying the translatome [41,42]. However, some pathophysiological processes, such as tumor angiogenesis, are improved by hypoxic stress and, in any case, protein synthesis remains robust. Therefore, the cell also exhibits Cap-independent mechanisms to support protein synthesis under stress conditions. For example, the internal ribosome entry sequence (IRES) in the 5′UTR of mRNAs can directly bind eIF4G without the eIF4E-mediation, allowing the assembly of ribosomes upstream of the start codon and promoting translation initiation through a Cap-independent mechanism. VEGFA mRNA has been reported to contain two IRESs in its 5′UTR and that these sequences up-regulate VEGFA expression in vivo under ischemic injury [38]. Notably, many pro-angiogenic factors, such as VEGF [43], FGF2 [44], Tie-2 [45] and HIF-1α [46], contain IRES.

**Table 1:** Molecular mechanism of mRNA translation regulation influenced by metabolic pathways during angiogenesis.

Main Translational Regulating Mechanism Involved	Molecular Pathway or Metabolic Stimulus	Molecular Mechanism of mRNA Translation Regulation	References
Cap-dependent translation regulation	MNK pathway	The MAPK-interacting kinases (MNKs) pathway induces eIF4E phosphorylation and promotes blood vessel growth.	[31]
	mTORC1 pathway	The mammalian target of rapamycin complex 1 (mTORC1) pathway induces hyperphosphorylation of 4EBP and angiogenesis.	[13,31]
Integrated stress response (ISR)	Amino acid deprivation	Amino acid restriction, in particular, that of sulfur-amino acids, triggers GCN2-ATF4 axis, eIF2α phosphorylation and angiogenesis.	[47,48]
Stress granule (SG) formation	Arsenite-induced oxidative stress	Arsenite treatment in ECs induces HIF-1α, up-regulating VEGF expression. In addition, it promotes SG formation in a YB-1-dependent manner.	[49,50]
	Energy deficiency	A drop in ATP levels due to low glycolysis or OXPHOS activity induces SG assembly.	[51]
tRNA modification	Amino acid deprivation	Uncharged tRNAs due to amino acid deficiency stimulate GCN2 to phosphorylate FBXO22, which, in turn, ubiquitinates mTOR at Lys2066 in a K27-linked manner.	[52]
		Arginine limitation regulates translation through ribosome pausing.	[53]
	Arsenite oxidative stress, heat shock or UV irradiation	Several stress stimuli induce angiogenin to cleave tRNAs into tRNA fragments.	[54]
		tRNAiMet fragments generated by AGO2 cleavage target PFKFB3, inhibiting angiogenesis.	[55]
		tRFGlnCTG targets 3′UTR of Antxr1.	[56]

## 3. Integrated Stress Response (ISR) in ECs During Tumorigenesis

Eukaryotic cells respond to stress stimuli through a common adaptive pathway known as the integrated stress response (ISR) (Figure 1). Several kinds of stress, such as nutrient deprivation or hypoxia, lead to the activation of the ISR [57]. This signaling cascade converges on a single molecular switch, which is the phosphorylation of eIF2α on serine residue 51 (P-eIF2α) [58]. The eIF2α kinase family includes general control non-derepressible 2 (GCN2), protein kinase dsRNA-dependent (PKR), PKR-like ER kinase (PERK), and heme-regulated inhibitor kinase (HRI) [59]. In response to stress conditions, these four serine/threonine kinases are activated through dimerization and autophosphorylation [60]. Meanwhile, eIF2α dephosphorylation is due to protein phosphatase 1 (PP1) complex formed by the PP1 catalytic subunit (PP1c) associated with the regulatory subunit GADD34 (growth arrest and DNA damage-inducible protein 34, or PPP1R15A) or CReP (constitutive repressor of eIF2α phosphorylation, or PPP1R15B) [58].

Upon P-eIF2α, the activity of the eIF2B guanine nucleotide exchange factor is impaired, preventing GDP-GTP exchange on the eIF2 γ-subunit and thus eIF2-GTP-tRNA_i_^Met^ ternary complex formation, resulting in the global down-regulation of Cap-dependent protein synthesis [61]. Concomitantly, the translation of specific mRNA transcripts containing upstream open reading frames (uORFs) or IRES in 5′UTR are up-regulated [62]. This selective translation, at the expense of the generalized one, leads to the production of ISR-effectors, such as activating transcription factor 4 (ATF4), and of ISR-regulators, such as GADD34, capable of inducing negative feedback on the ISR pathway. Global translation arrest due to eIF2α phosphorylation and ATF4-mediated induction of 4EBP [63] allows the cell to survive the stress until its resolution or to die by apoptosis. If the stress lasts too long, the ISR can promote apoptosis through the overexpression of death receptors [64,65] or via GADD34 [66]. Thus, the ISR plays a dual role, protecting the cell from the effects of stress but triggering apoptosis once the threshold limit is exceeded.

Metabolic conditions, such as nutrient deprivation, amino acid [47] or glucose [65] deficiency, can trigger ISR. Given the central role of the ISR pathway in regulating protein synthesis, it is not surprising that the limitation of amino acids, which are the building blocks of proteins, induces the ISR. The response to amino acid starvation is predominantly mediated by the GCN2-ATF4 axis [67,68,69], and the ATF4 knockout impairs amino acid uptake, sensitizing cells to oxidative stress [47]. The antioxidant role of amino acid metabolism is linked to the biosynthesis of glutathione (GSH), the most abundant reducing agent in cells. GSH is a tripeptide composed of glutamate (Glu), cysteine (Cys) and glycine (Gly) residues, and its ROS scavenging action is achieved by the thiol group (-SH) on the Cys-residue. In fact, Cys starvation induces ferroptosis [70], a cell death characterized by a drop in GSH levels, an increase in ROS levels and the peroxidation of lipid membranes. ISR up-regulates enzymes that lead to cysteine synthesis, such as cystathionine γ-lyase (CTH) [71], or to the import of cysteine dimers (cystine, Cys-Cys), such as the SLC7A11 transporter, through the ATF4-mediated induction of NRF2 [72]. In addition, the increased levels of hydrogen sulfide (H_2_S), due to the ATF4-induced CTH enzyme, play an antioxidant role through persulfidation [73]. Interestingly, sublethal levels of oxidative stress promote the expression of pro-angiogenic factors such as VEGF [49]. VEGF is a transcriptional target of ATF4 [74], and its expression can be induced by nutrient restriction [75]. The formation of new blood vessels is associated with the up-regulation of VEGF and a specialized pro-survival antioxidant translation program. Cells that have high metabolic and energetic demands and thus high ROS production, such as proliferating cells, escape cell death through the expression of the antioxidant translation program. In fact, ISR caused by sulfur-amino acid deprivation triggers angiogenesis [48] (Table 1). ATF4-mediated ISR promotes angiogenesis in both physiological [76] and pathological (tumor angiogenesis) contexts [77,78,79]. By evaluating the stress response landscape in the tumor microenvironment (TME) of different carcinomas (breast, pancreas, ovary and prostate), Lior and colleagues reported that stress responses of non-immune stromal cells are highly activated in the proximity of cancer cells. Furthermore, through single-cell transcriptomic analysis, they observed that endothelial cells have a stronger response to hypoxia stress than other stromal cells, while pericytes and fibroblasts are associated with NRF2-mediated oxidative stress response [80].

Notably, metabolic programming is a cancer hallmark, and the dysregulation of the ISR pathway allows tumor cells to adapt to stress conditions. In some tumor subtypes, such as pancreatic cancer, constitutive activation of the ISR has been observed [81], while in some others, such as prostate cancer, the ISR-regulator GCN2 maintains amino acid homeostasis [82]. ISR inhibition sensitizes tumor cells to chemotherapy treatments [83]. Therefore, ISR attenuation may represent an additional strategy to target tumor vasculature [84,85].

## 4. Stress Granule Formation and Translatome Remodeling in Tumor Context

Under stress conditions, eIF2α phosphorylation and ISR activation block global translation. Instead of disassembling the translation machinery in an energy- and time-consuming process, the cell sequesters non-translating messenger ribonucleoprotein particles (mRNPs), growth factors and pro-apoptotic factors in cytoplasmic organelles until the stressor disappears [86]. These organelles, known as stress granules (SGs) (Figure 1), are membraneless foci generated by liquid–liquid phase separation (LLPS), in which RNAs and proteins are temporarily accumulated [87]. SG assembly is a eukaryotic conserved adaptive mechanism extensively investigated in the neurodegenerative field, which is also emerging in cancer studies as a defense mechanism against the stress induced by the tumor microenvironment.

Structurally, SGs are formed by a stable core component surrounded by a more dynamic shell containing a lower density of RNPs. SG assembly is orchestrated by RNA-binding proteins such as G3BP1 (Ras-GTPase-activating protein SH3 domain-binding protein 1) [88] or TIA-1 (T-cell internal antigen-1) [89]. Interestingly, P-eIF2α-independent SG formation has been reported to occur due to the deregulation of translation initiation factors, suggesting that the inhibition of ribosome assembly is the major trigger for SGs [90,91,92]. Another non-canonical pathway for SG formation is due to energy deficiency [51]. It has been found that in high glucose-consuming cells, such as brain cells, glycolysis and mitochondrial oxidative phosphorylation (OXPHOS), but not the pentose phosphate pathway (PPP), were required to maintain the intracellular ATP pool and prevent SG formation [51] (Table 1). In addition to neurons, proliferating ECs are also highly dependent on glucose catabolism to sustain their energy demands [11]. However, although SG assembly has been observed in both ECs [50] and vascular smooth muscle cells (VSMCs) [93], the role of SGs in tumor vasculature remains poorly investigated. Nevertheless, treatment with sodium arsenite, a widely used oxidative stress drug to induce SGs, leads to the up-regulation of VEGF [49] and SG assembly [94] (Table 1). In particular, recent evidence has shown that the down-regulation of the tight junction protein zonula occludens-1 (ZO-1) under arsenite-induced oxidative stress conditions enhances the formation of SGs in a Y-box binding protein 1 (YB-1)-dependent manner in ECs [50]. YB-1 is a DNA- and RNA-binding protein involved in SG assembly through the increased post-transcriptional expression of G3BP1 [94]. Moreover, YB-1 knockdown in a tumor xenograft model inhibited tumor angiogenesis and reduced the expression of VEGFR2 and Tie-2 receptors [95]. This suggests that YB-1 is sequestered at cell–cell junctions by ZO-1, but under stress conditions, the disruption of these junctions allows the release of YB-1, resulting in the formation of SGs.

SG formation promotes cell survival by inhibiting pro-apoptotic kinases [96] and reducing ROS production [97]. However, in many cases, the molecular mechanisms underlying this pro-survival effect remain unknown. Prolonged nutrient starvation is a physiological SG trigger that induces metabolic reprogramming in cells. Under nutrient starvation, lipid consumption through fatty acid β-oxidation (FAO) drives an increase in ROS that can damage cellular structures [98]. Recently, it has been shown that SG formation attenuates FAO through a reduction in mitochondrial permeability caused by the depletion of voltage-dependent anion channels (VDACs), fatty acid importers into mitochondria [99]. However, the relationship between SG and FAO may be more complicated in ECs. Indeed, on the one hand, FAO is necessary to support angiogenesis [100]; on the other hand, SG formation also seems to play a pro-angiogenic role in ECs [50]. Further investigations are needed to shed light on the role of SGs in controlling EC metabolism during tumor angiogenesis.

Understanding the interactions between SG biology and angiogenesis could have therapeutic implications. For example, in solid tumors following ionizing photon radiotherapy, it is possible to observe a resumption of angiogenesis [101] with consequent cancer relapse. This is due to the activation of the HIF-1α pathway, which leads to the expression and release of VEGF, which in turn confers radioresistance to tumor endothelial cells [102,103]. By interfering with SG assembly through G3BP1 silencing, it is possible to radiosensitize cancer cells and induce cell death by excessively increasing ROS levels [104]. A second example comes from cardiovascular diseases; atherosclerosis is a chronic inflammatory disease in which a lesion in the arterial wall leads to the formation of oxidized low-density lipoprotein (ox-LDL) plaques that obstruct the lumen of the vessel. In two distinct animal models of atherosclerosis, i.e., *LDLR*−/− or *ApoE*−/− mice, SG markers such as G3BP1/2 and PABP (poly-A binding protein) were correlated with atherosclerotic lesions. Furthermore, the knockdown of G3BP1/2 reduces SG formation, attenuates inflammation and decreases lesion amount [93,105].

## 5. Impact of Metabolic tRNA Modifications on Tumor Angiogenesis

Since their discovery in 1958, the involvement of transfer RNAs (tRNAs) in protein synthesis has been clear [106] (Figure 1). In fact, their primary role consists of the transport of single amino acids to the ribosome to extend the nascent polypeptide chain. Therefore, they are key molecules in the passage of genetic information from a nucleic acid matrix to a peptide form. In general, these small non-coding RNAs (76–90 nt) have a two-dimensional cloverleaf structure consisting of an acceptor stem, a D-arm, an anticodon arm, a variable length arm and a T-arm (TΨC arm). However, three-dimensionally, tRNAs exhibit a conserved L-shaped structure at the ends of which are the anticodon and the amino acid acceptor [107]. Intramolecular interactions between the acceptor stem and the T-arm constitute the acceptor domain, which is recognized by the eukaryotic elongation factor EF1A, while the anticodon domain is formed by the anticodon arm and the D-arm, and the two domains meet at the elbow. The enzymatic family of aminoacyl-tRNA synthetases (ARSs) esterify an amino acid to the 3′ acceptor CCA of tRNA. Each ARS is specific for one amino acid, 20 canonical proteinogenic amino acids and the non-canonical selenocysteine and a tRNA iso-acceptor group. Iso-acceptors are pools of different tRNAs capable of loading the same amino acid. Charging the amino acid onto the tRNA is a two-step reaction that begins with the condensation of the amino acid and an ATP molecule into an aminoacyl-adenylate, and then the amino acid is transferred to the 2′- or 3′-hydroxyl groups of the terminal adenosine ribose of a tRNA [108].

In response to amino acid starvation, tRNAs trigger the ISR. In yeast, uncharged tRNAs accumulated during amino acid starvation bind to the histidyl-tRNA synthetase (HisRS)-like domain of GCN2, enhancing eIF2α phosphorylation activity [109,110]. A novel nutrient sensing mechanism based on deacylated tRNA-GCN2 interaction to inhibit mTOR activity has recently been described [52] (Table 1). Furthermore, in mammals, it has been observed that some uncharged tRNAs can regulate translation through ribosome pausing [53], which in turn induces the activation of GCN2 [111].

Mature tRNAs can give rise to several small non-coding RNAs through cleavage into tRNA fragments (tRFs) [112]. Specific stress-induced tRFs have been observed. For example, under stress conditions, angiogenin (ANG), a ribonuclease that stimulates the formation of new blood vessels, cleaves tRNAs [54,113]. The tRNA-derived stress-induced RNAs (tiRNAs) inhibit protein synthesis [54,114]. In particular, 5′- but not 3′-tRNA fragments (5′-tiRNA) induce SG assembly via YB-1 [115] and block global translation. In addition to their role in translation, tRNA fragments can regulate gene expression by promoting the degradation of target mRNAs [116] and inducing epigenetic changes [117]. Although the functions of tRFs are still poorly understood, their use in clinical practice could bring some benefits [118]. tRFs usually act as micro-RNA (miRNA), targeting complementary sequences. Recently, some of these fragments have been implicated in the regulation of pathological angiogenesis. The 31-nt tRF^Met^ generated by the argonaute 2 (Ago2)-induced cleavage of translation initiator aminoacyl-tRNA (tRNA_i_^Met^) blocks angiogenesis in breast cancer by targeting the glycolytic enzyme 6-phosphofructo-2-kinase/fructose-2,6-bisphosphatase 3 (PFKFB3) [55], while tRF^Gln^ reduces angiogenesis by inhibiting Antxr1 [56], a tumor angiogenesis marker. Interestingly, tRF^Val^ and tRF^Gly^ generated by angiogenin cleavage have been found to reduce angiogenesis in a rat brain ischemic model. Thus, tRFs may induce negative feedback on angiogenesis triggers by ischemic injury [119].

In addition to structural changes due to proteolytic cleavage, it should be kept in mind that tRNAs are often chemically modified. On average, each eukaryotic tRNA contains 13 modifications that alter its biological role. To date, hundreds of modified tRNA bases have been recorded in the MODOMICS database (https://iimcb.genesilico.pl/modomics/, accessed on 17 October 2024) [120]. Chemical modification of the bases in response to environmental conditions, frequently through methylation, can impact tRNA stability and translation efficiency. For example, low-glucose conditions enhance ALKBH1 expression, a N^1^-methyladenosine (m^1^A, usually at A58) tRNA eraser, attenuating translation initiation and reducing the use of tRNAs in protein synthesis [121]. 5-methylcytosine (m^5^C) at C38 of tRNAs, induced by the m^5^C38 writer Dnmt2, prevents the fragmentation of tRNAs, and its impairment leads to amino acid misincorporation during translation in the bone marrow [122], while m^5^C into the wobble-uridine nucleotide (U34) of mitochondrial tRNA^Met^ promotes the synthesis of OXPHOS proteins [123]. It has also been reported that, in yeast, the intracellular concentration of sulfur-containing amino acids, such as methionine and cysteine, controls the thiolation of the U34 in lysine, glutamine, and glutamate tRNAs [124]. The fact that point mutations in tRNAs can alter blood vessel formation [125] and that the metabolic state of the cell can lead to the modification of the tRNA bases suggests that tRNAs and their modifications may play a crucial role in the control of angiogenesis.

## 6. Conclusions and Future Perspectives

The survival rate for cancer patients is dependent upon innovative anti-angiogenic therapies and demands the development of alternative and complementary therapies to the existing ones. In this sense, it is known that protein synthesis and its control are central to biochemical signature. The maintenance of protein homeostasis is an energy-expensive process that needs the coordinated regulation of transcription and translation [126]. Although changes in mRNA expression play an obvious role in determining the cellular levels of proteins, several studies show that there is not a strict correlation between these two phenomena, with some abundant mRNAs translated poorly and vice versa. Generally, the number of ribosomes translating a specific mRNA is the most critical parameter of the rate of synthesis of a specific protein [127]. However, the process by which the cancer-induced metabolic rewiring of TECs [128] modulates the mRNA translational machinery to support vessel formation is elusive. Conversely, it is unknown whether the modulation of the mRNA translational machinery could remodel the metabolic program of ECs during tumor progression. It is necessary to gain a deeper understanding of how tumor vessel mRNA translation is altered in specific cancer types and how the vessel phenotype can be modulated by the translational machinery. This knowledge may lead to new vascular targeting strategies that either directly target the tumor vessels or optimize their function to fit the cancer therapy at hand.
